# Supplementary material for: Corrosion engineering towards efficient oxygen evolution electrodes with stable catalytic activity for over 6000 hours
Source: Nat Commun. 2018 Jul 4;9:2609. doi: 10.1038/s41467-018-05019-5 (PMC6031686; doi:10.1038/s41467-018-05019-5)
Supplement: Supplementary file 1 — Supplementary Information [file 41467_2018_5019_MOESM1_ESM.pdf]

Supplementary Information (SI) for:

Corrosion Engineering towards Efficient Oxygen Evolution Electrodes with Stable Catalytic Activity for over 6000 Hours

Yipu Liu,[a] Xiao Liang,[a] Lin Gu,[b] Yu Zhang,[c] Guo-Dong Li,[a] and
Xiaoxin Zou[a,*] Jie-Sheng Chen[d]

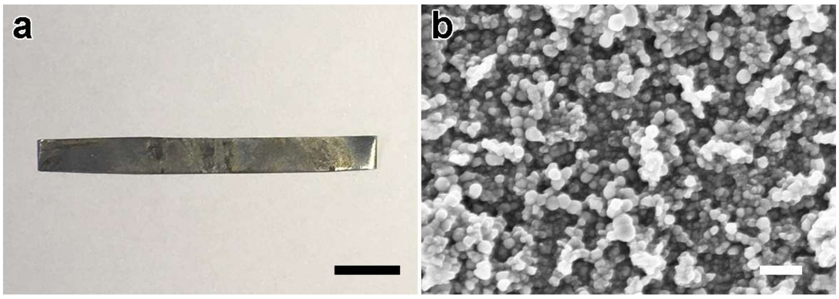

Supplementary Figure 1. Structural characterization of the corroded iron plate. a, Digital image and **b**, SEM image of an iron plate that was corroded in an aqueous solution without divalent ions at room temperature for 12 hours. Scale bars, 1 cm and 400 nm in **a** and **b**, respectively.

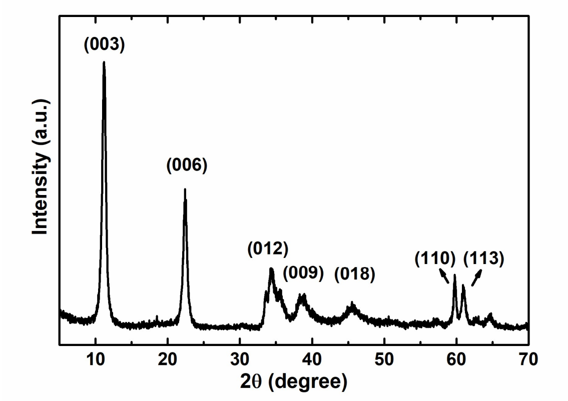

Supplementary Figure 2. XRD characterization of powdered LDH material. XRD pattern of powdered NiFe-LDH synthesized from a conventional hydrothermal method.[1]

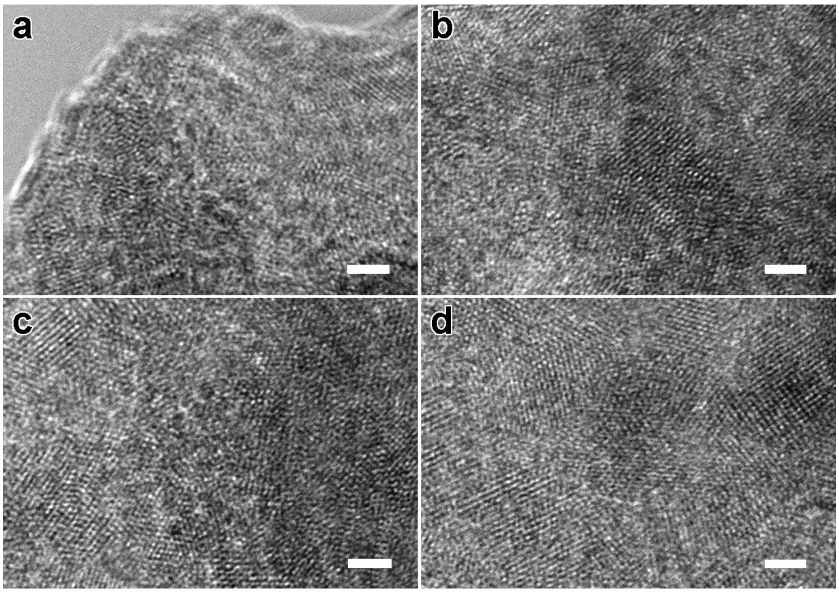

Supplementary Figure 3. HRTEM characterization. a-d, HRTEM images of O_2-Cat-1. Scale bars, 2 nm in **a**, **b**, **c** and **d**, respectively.

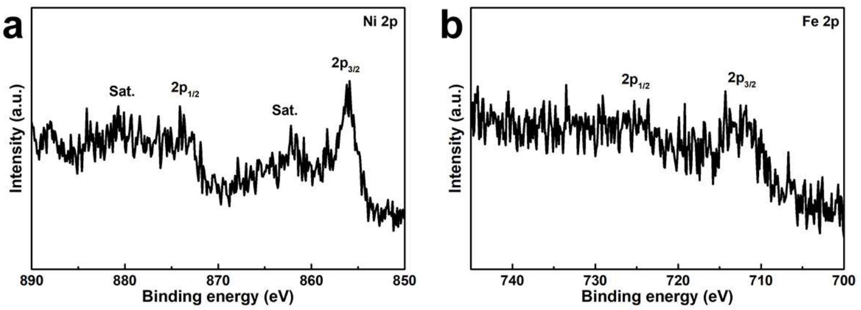

Supplementary Figure 4. XPS characterization. **a**, Ni 2p and **b**, Fe 2p core level XPS spectra of O_2-Cat-1.

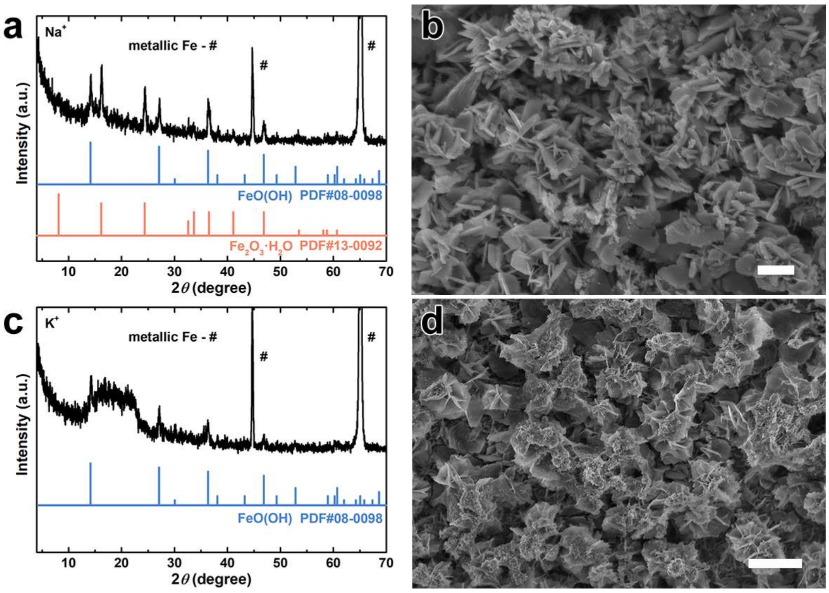

Supplementary Figure 5. XRD and SEM characterizations. **a**, XRD pattern and **b**, SEM image of the sample obtained by the corrosive treatment of iron plate in the aqueous solution containing Na^+ cations. **c**, XRD pattern and **d**, SEM image of the sample obtained by the corrosive treatment of iron plate in the aqueous solution containing K^+ cations. Scale bars, 1 μm and 10 μm in **b** and **d**, respectively.

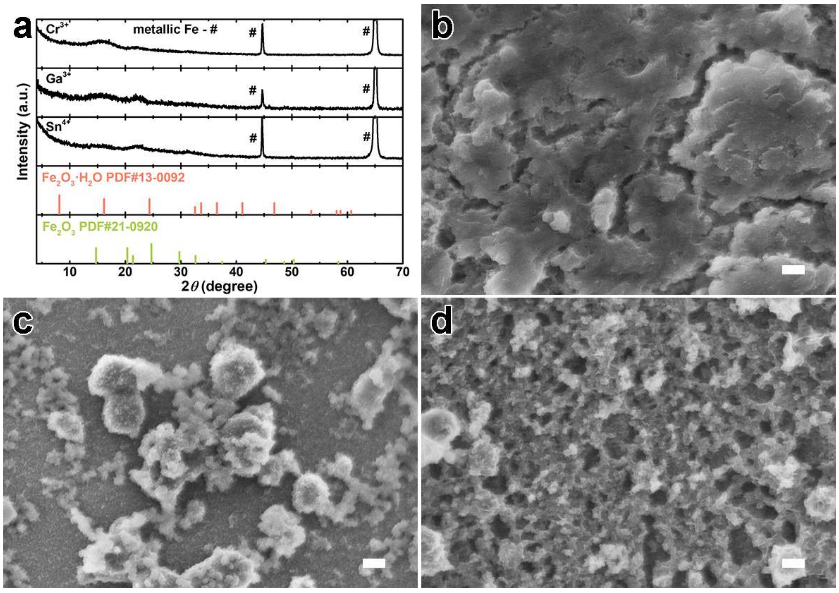

Supplementary Figure 6. XRD and SEM characterizations. **a**, XRD patterns and **b-d**, SEM images of the samples obtained by the corrosive treatment of iron plate in the aqueous solution containing Cr^{3+}, Ga^{3+} or Sn^{4+} cations. Scale bars, 200 nm in **b**, **c** and **d**, respectively.

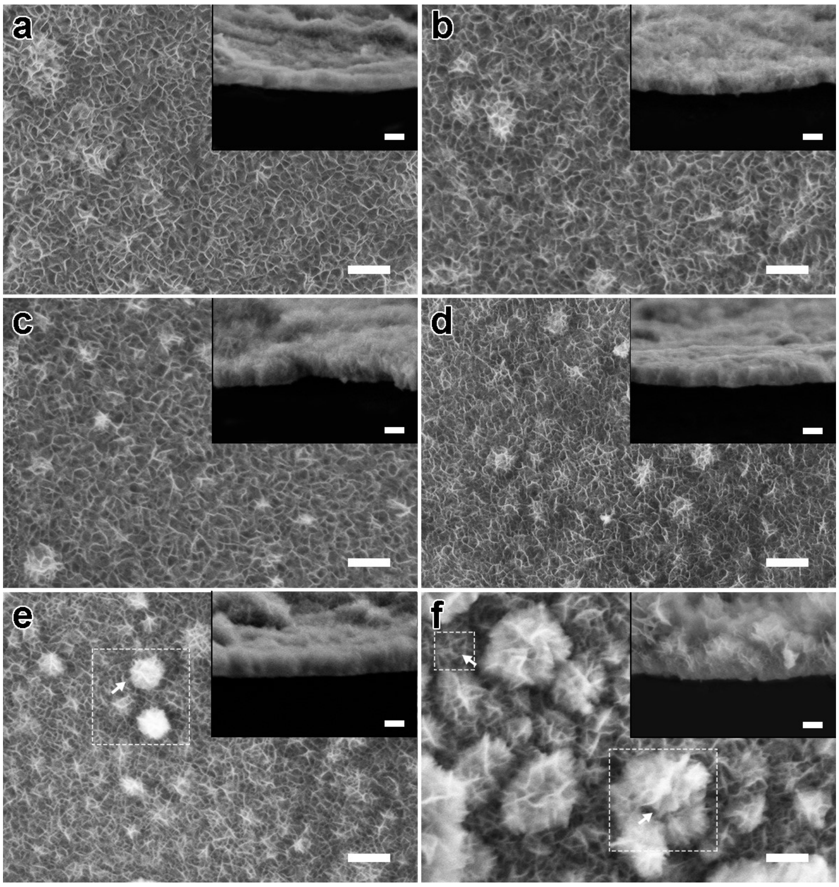

Supplementary Figure 7. SEM characterization of a series of iron-plate-derived samples. SEM images of the samples obtained by the corrosive treatment of iron plate in the aqueous solution containing **a**, 200 mmol/L, **b**, 80 mmol/L, **c**, 60 mmol/L, **d**, 45 mmol/L, **e**, 20 mmol/L, **f**, 10 mmol/L Ni^{2+} cations. Scale bars, 400 nm in **a**, **b**, **c**, **d**, **e** and **f**, respectively, and 200 nm in the corresponding inset.

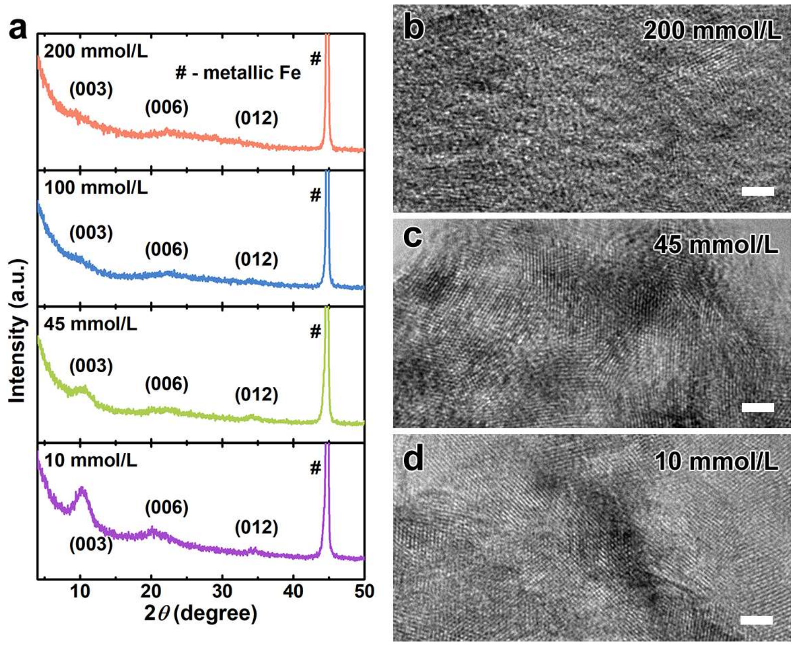

Supplementary Figure 8. XRD and HRTEM characterizations. a, XRD patterns and **b-d**, HRTEM images of the materials obtained by the corrosive treatment of iron plate in the aqueous solution containing **b**, 200 mmol/L, **c**, 45 mmol/L, **d**, 10 mmol/L Ni^{2+} cations. The results reveal that the materials obtained from the solutions containing higher concerntration of Ni^{2+} cations comprise the LDH thin films with a lower crystallinity. Scale bars, 2 nm in **b**, **c**, and **d**, respectively.

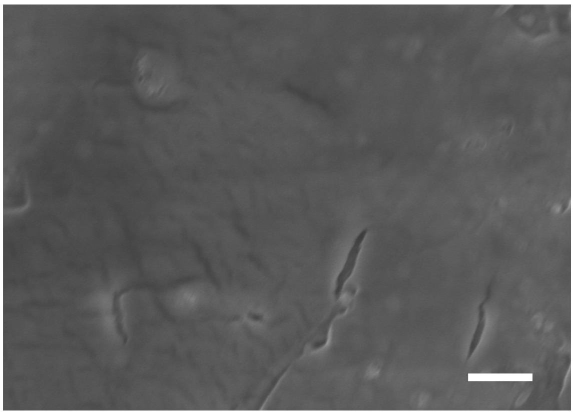

Supplementary Figure 9. SEM characterization of the sample obtained in the absence of air. SEM image of an iron plate that was corroded in an aqueous solution containing divalent ions, yet without air, at room temperature for 12 hours. Scale bar, 400 nm.

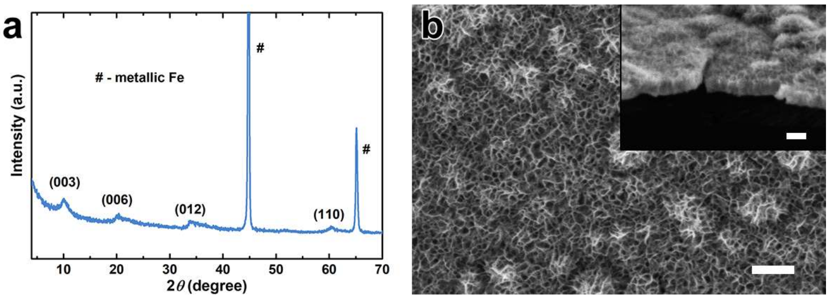

Supplementary Figure 10. XRD and SEM characterizations of the sample obtained in the absence of CO_2. (a) XRD pattern and (b) SEM image of the material obtained by the corrosive treatment of iron plate in the aqueous solution containing 100 mmol/L Ni^{2+} ions under a pure oxygen atmosphere. Scale bars, 400 nm in **b** and 200 nm in the inset. Characteristic peaks of LDH can be identified from the XRD pattern, and an interlayer spacing of ca. 0.862 nm, attributable to the LDHs with SO_4^{2-} as the intercalated anions[2], can be calculated from the (003) diffraction peak of the material. The above result suggests that when CO_2/CO_3^{2-} is absence, the SO_4^{2-} anions in the aqueous solution may serve as charge compensating anions between the layers of the as-formed LDH, instead of carbonate, to maintain the charge balance of the whole structure.

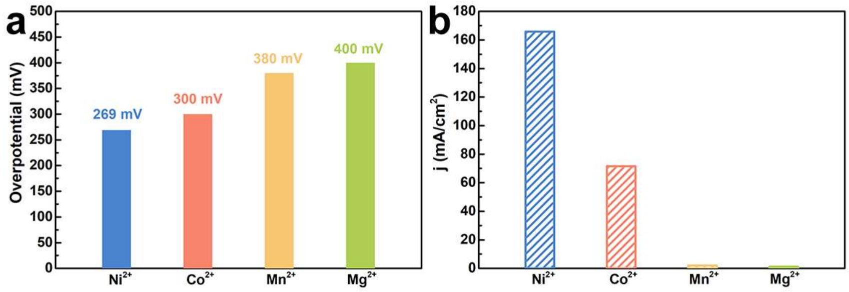

Supplementary Figure 11. Activity comparison of the different LDH materials. Comparison of **a**, overpotentials at a current density of 10 mA/cm^2 and **b**, current densities at a potential of 1.78 V *vs* RHE among materials obtained by the corrosive treatment of iron plate in the aqueous solution containing Ni^{2+}, Co^{2+}, Mn^{2+} or Mg^{2+} cations. The result reveals that the material obtained in the corrosive environment containing Ni^{2+} shows the best electrocatalytic activity for OER.

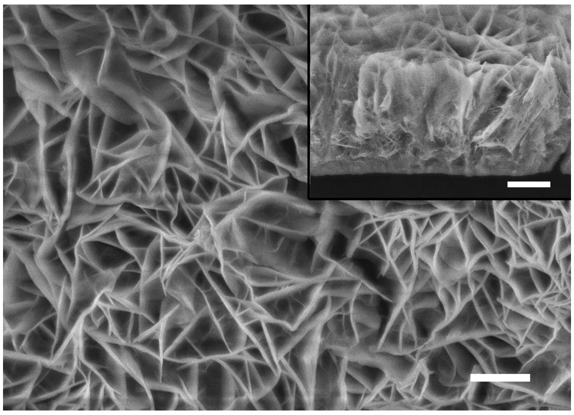

Supplementary Figure 12. SEM characterization. Top-down SEM image of O_2-Cat-2 with corresponding cross-section SEM image shown in the inset. Scale bars, 400 nm and 1 μm (inset), respectively.

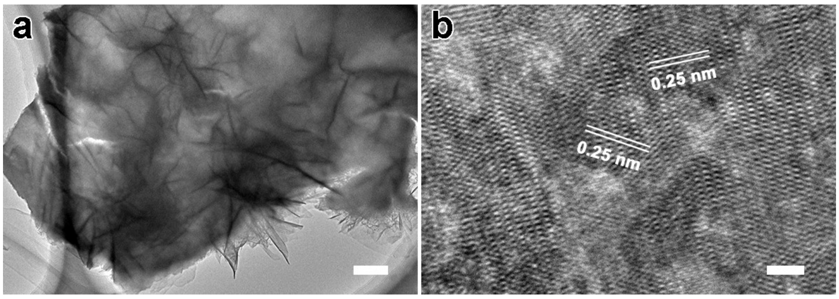

Supplementary Figure 13. TEM and HRTEM characterizations. **a**, TEM and **b**, HRTEM images of O_2-Cat-2. The characterization results reveal that the LDH nanosheets in O_2-Cat-2 comprise larger crystalline domains relative to O_2-Cat-1. Scale bars, 500 nm and 2 nm in **a** and **b** respectively.

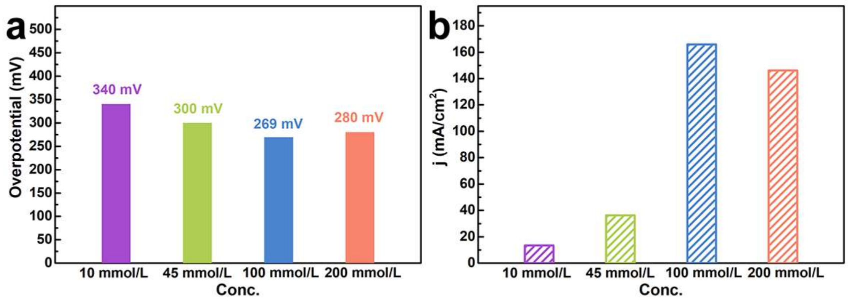

Supplementary Figure 14. Activity comparison of the different LDH materials. Comparison of **a**, overpotentials at a current density of 10 mA/cm^2 and **b**, current densities at a potential of 1.78 V *vs* RHE among materials obtained by different concentration of Ni^{2+} cations as corrosion environment.

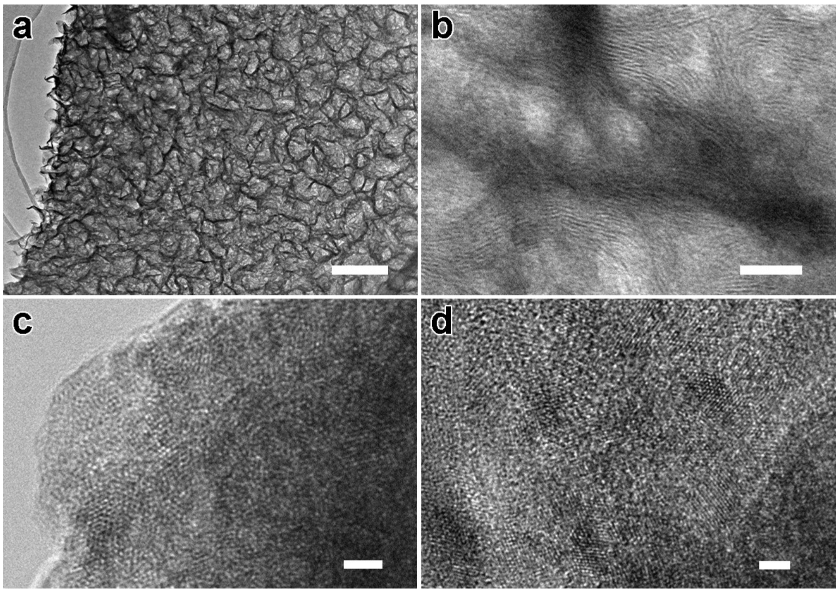

Supplementary Figure 15. Structural characterization of the material after OER. **a**, TEM and **b-d**, HRTEM images of O_2-Cat-1 after 100 h-long electrocatalysis test. Scale bars, 200 nm, 10 nm, 2 nm and 2 nm in **a**, **b**, **c** and **d**, respectively.

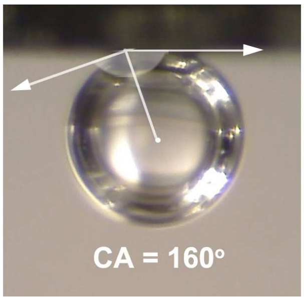

Supplementary Figure 16. Adhesion property of gas bubbles. The underwater bubble contact angle (160 °) of O_2-Cat-1. The underwater bubble contact angle (CA) of O_2-Cat-1 is measured to be ~160°, indicative of the underwater superaerophobic property (CA > 150°) of O_2-Cat-1.[3,4] The measurement was carried out according to the experimental previously-reported procedures.[2] The property is generally considered to be associated to the nanoarray structure and has been proven to be beneficial for the evacuation of the gases generated during electrochemical reactions.[2]

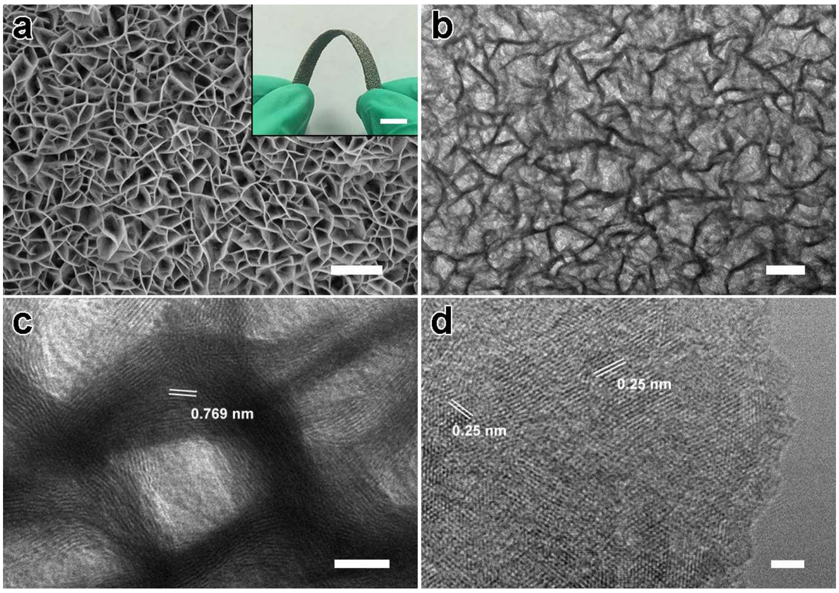

Supplementary Figure 17. Structural characterization of a 3D electrode. a, SEM image of 3D-O_2-Cat-1 with corresponding digital image shown in the inset. Scale bars, 500 nm in **a** and 1 cm in the inset. **b**, TEM and **c-d**, HRTEM images of 3D-O_2-Cat-1. Scale bars, 100 nm, 10 nm and 2 nm in **b**, **c** and **d**, respectively.

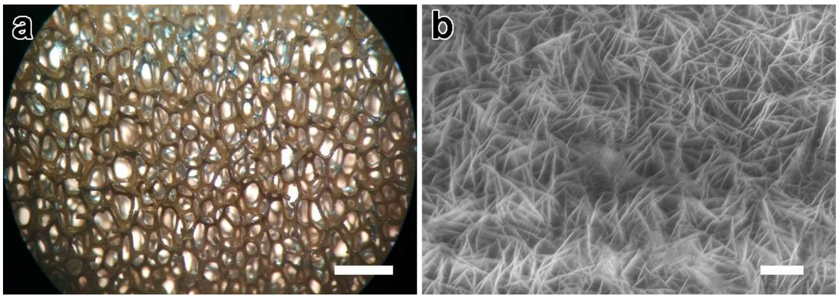

Supplementary Figure 18. Morphology of a 3D electrode. a, Digital and **b**, SEM images of 3D-O_2-Cat-2. Scale bars, 1 mm and 400 nm in **a** and **b**, respectively.

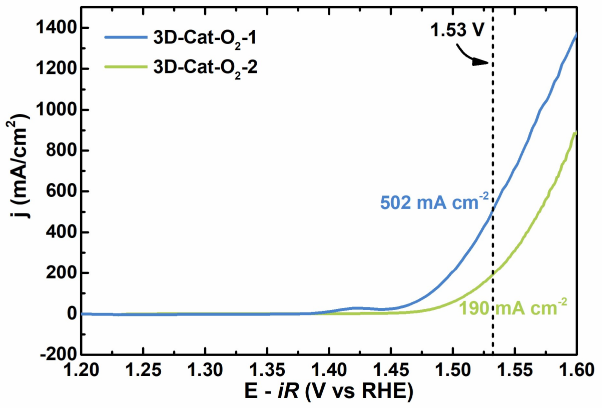

Supplementary Figure 19. Activity comparison between two 3D electrodes. Comparison of electrochemical performance between 3D-O_2-Cat-1 and 3D-O_2-Cat-2.

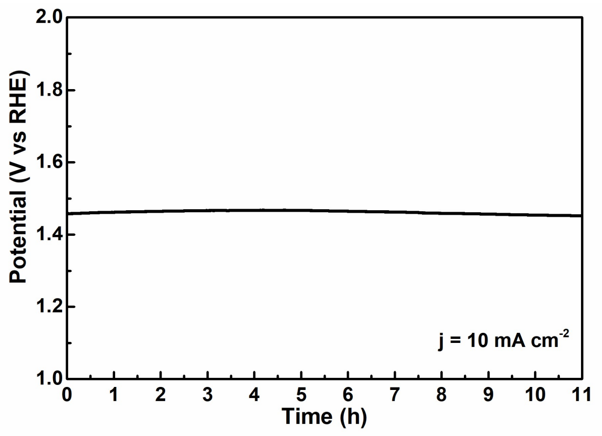

Supplementary Figure 20. Stability at small current density. Chronopotentiometric curves of 3D-O_2-Cat-2 in 1 M KOH at a current density of 10 mA/cm^2 (without *iR*-correction).

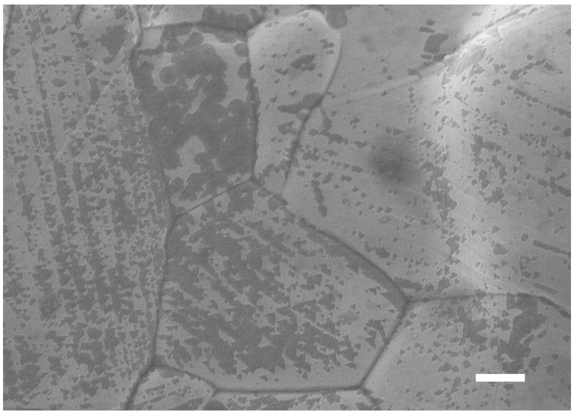

Supplementary Figure 21. Structural instability at large current density. SEM image of 3D-O_2-Cat-2 after the stability test conducted under a current density of 1000 mA/cm^2. Scale bar, 2 μm. The result reveals that the LDH nanosheet arrays peeled off from the nickel foam during the electrocatalysis.

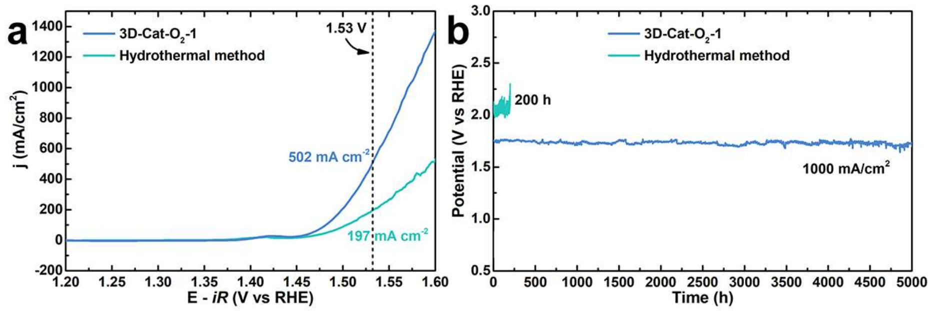

Supplementary Figure 22. Catalytic activity and stability between two 3D electrodes. Comparison of **a**, OER activity and **b**, stability between 3D-O_2-Cat-1 and the LDHs-based electrode fabricated by the hydrothermal deposition method in 1 M KOH at a current density of 1000 mA/cm^2 (without *iR*-correction).

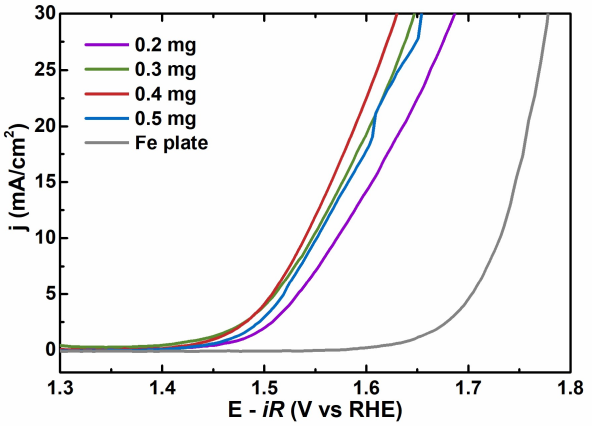

Supplementary Figure 23. Activity with different IrO_2 loading. *iR*-corrected (85%) polarization curves of IrO_2 in 1 M KOH electrolyte with different loading amounts (0.2- 0.5 mg).

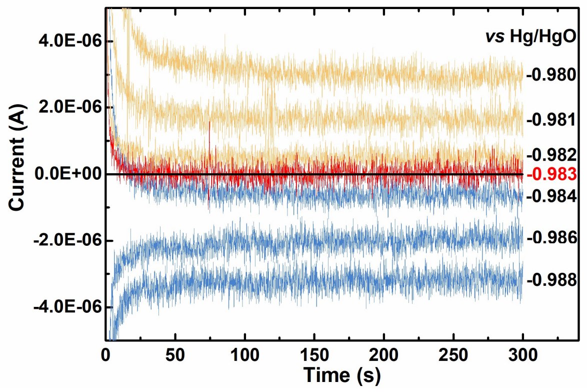

Supplementary Figure 24. Calibration of reference electrode. Representative 300 s potential-controlled chronoamperometric curves from -0.988 V to -0.980 V *versus* Hg/HgO electrode in H_2-saturated 10 M KOH.

Supplementary Table 1. Comparison of the electrocatalytic performance of O_2-Cat-1 and 3D-O_2-Cat-1 with some representative electrodes (or electrocatalysts) reported recently for OER.

Catalyst	Electrolyte solution	Substrate	Overpotential at *j* =10 mA/cm^2	Stability test	Reference
**O_2-Cat-1**	1 M KOH	Fe plate	269 mV	100 mA cm^{-2}@100 h	This work
**3D-O_2-Cat-1**	1 M KOH	Fe foam	200 mV	1000 mA cm^{-2}@6000 h	This work
LDH-based materials:					
**Water-plasma exfoliated CoFe-LDH nanosheet**	1 M KOH	Ni foam	232 mV	20 mA cm^{-2}@11 h	*Adv. Mater.* **2017**, 29, 1701546.
**NiFe-LDH-NS@DG10**	1 M KOH	Ni foam	200 mV	10 mA cm^{-2}@10 h	*Adv. Mater.* **2017**, 29, 1700017.
**Exfoliated NiCo-LDH nanosheets**	1 M KOH	Carbon paper	367 mV	20 mA cm^{-2}@6 h	*Nano Lett.* **2015**, 15, 1421.
**NiFe-LDH/NF**	1 M KOH	Ni foam	224 mV	225mA cm^{-2}@50 h	*Chem. Sci.* **2015**, 6, 6624.
**NiFe-LDH/NF**	1 M KOH	Ni foam	240 mV	30 mA cm^{-2}@10 h	*Science* **2014**, 345, 1593.
**Exfoliated NiFe-LDH nanosheets**	1 M KOH	GCE	300 mV	10 mA cm^{-2}@13 h	*Nat. Commun.* **2014** , 5 ,4477.
**3D-NiFe-LDH film**	1 M KOH	Ni foam	300 mV	200 mA cm^{-2}@10 h	*Chem. Commun.* **2014**, 50, 6479
**NiFe-LDH/CNTs/CFP**	1 M KOH	Carbon fiber paper	240mV	20 mA cm^{-2}@1 h	*J. Am. Chem. Soc.* **2013**, 135, 8452.
Other representative matrials:					
**WCoFe oxyhydroxides**	1 M KOH	Ni foam	250 mV	20 mA cm^{-2}@~500 h	*Angew.Chem.Int. Ed.* **2017**, 56, 4502.
**Gelled FeCoW oxyhudroxides**	1 M KOH	Au-plated Ni foam	191 mV	30 mA cm^{-2}@550 h	*Science* **2016**, 352, 333.
**Li-induced ultra-small NiFeO$_x$ nanoparticle**	1 M KOH	Carbon fiber paper	280 mV	10 mA cm^{-2}@200 h	*Nat Commun.* **2016**, 6, 7261.
**$NiCo_2O_4$ hollow microcuboids**	1 M NaOH	Ni foam	290 mV	10 mA cm^{-2}@30 h	*Angew.Chem.Int. Ed.* **2016**, 55, 6290.
**Double perovskite $PrBa_{0.5}Sr_{0.5}Co_{1.5}Fe_{0.5}O_{5+\delta}$**	0.1 M KOH	GCE	350 mV	10 mA cm^{-2}@2 h	*Nat Commun.* **2017**, 8, 14586.
**Double perovskiets $(Pr_{0.5}Ba_{0.5})CoO_{3-\delta}$**	0.1 M KOH	GCE	~330 mV	5 mA cm^{-2}@2 h	*Nat Commun.* **2013**, 4, 2439.

Supplementary Table 2. Ion concentration of iron and nickel in electrolyte during the long-time electrolysis process at 1000 mA/cm^2 in the presence of 3D-O_2-Cat-1.

Time (h)	iron ion concentration (mol/L)	%RSD[a] (iron)	nickel ion concentration (mol/L)	%RSD (nickel)
2	0	91.14	0	21.99
4	0	26.79	0	0.94
6	0	169.1	0	0.32
8	0	67.13	0	13.1
10	7.14×10^{-7}	525.4	0	37.98
34	0	33.2	0	16.36
58	3.57×10^{-7}	108.3	0	7.55

[a]: RSD is related to the relative standard deviation.

Supplementary References

(1) Shao, M.; Wei, M.; Evans, D.G.; Duan, X. *Chem. Commun.* **2011**, *47*, 3171.
(2) Hunter, B. M.; Hieringer, W.; Winkler, J. R.; Gray, H. B.; Müller, A. M. *Energy Environ. Sci.* **2016**, *9*, 1734.
(3) Lu, Z.; Zhu, W.; Yu, X.; Zhang, H.; Li, Y.; Sun, X.; Wang, X.; Wang, H.; Wang, J.; Luo, J.; Lei, X.; Jiang, L. *Adv. Mater.* **2014**, *26*, 2683.
(4) He, J.; Hu, B.; Zhao, Y. *Adv. Funct. Mater.* **2016**, *26*, 5998.
