# Supplementary material for: Corrosion engineering towards efficient oxygen evolution electrodes with stable catalytic activity for over 6000 hours
Source: Nat Commun. 2018 Jul 4;9:2609. doi: 10.1038/s41467-018-05019-5 (PMC6031686; doi:10.1038/s41467-018-05019-5)
Supplement: Supplementary file 3 — Description of Additional Supplementary Files [file 41467_2018_5019_MOESM3_ESM.pdf]

Description of Additional Supplementary Files

File Name: Supplementary Movie 1
Description: The movie showing the stable oxygen evolution reaction on O2-Cat-1 at large current density.
